# Differential Effects of Cardiac Rehabilitation in Obese and Non-Obese Population

**DOI:** 10.7759/cureus.18227

**Published:** 2021-09-23

**Authors:** Varunsiri Atti, Pradeep Kumar Devarakonda, Sameer Raina

**Affiliations:** 1 Cardiology, West Virginia University Heart and Vascular Institute, Morgantown, USA; 2 Internal Medicine, The Brooklyn Hospital Center, Brooklyn, USA

**Keywords:** cardiac rehabilitation, phase-i cardiac rehabilitation, chronotropic competence, metabolic equivalents, blood pressure response, cardiology, cardiology research, internal medicine, obesity, extreme obesity

## Abstract

Background

Cardiac rehabilitation (CR) improves outcomes in patients with heart disease. We investigated the differences in outcomes of comprehensive phase II CR in obese and non-obese patients.

Methods

We performed a retrospective analysis of functional outcomes including metabolic equivalents (METS), heart rate (chronotropic competence - CC), and blood pressure response (BPR) in 178 patients undergoing CR based on underlying body mass index (BMI). Demographic and clinical variables were assessed for age, gender, race, smoking, hypertension, hyperlipidemia, diabetes mellitus, coronary artery disease, stroke, heart failure, medication use, and several sessions attended.

Results

Initial CC and METS were impaired in majority of patients attending CR, whereas BPR to exercise was mostly preserved. Significant improvement occurred in CC (non-obese: 0.71 ± 0.11 vs 0.76 ± 0.11, p < 0.001; obese: 0.72 ± 0.10 vs 0.75 ± 0.12, p = 0.0010) and METS (non-obese: 4.96 ± 1.98 vs 7.33 ± 2.94, p < 0.001; obese: 4.39 ± 1.81 vs 6.79 ± 3.34, p < 0.001). Post-CR obese patients were able to reach similar level of physical activity as non-obese patients (6.79 ± 3.34 vs 7.33 ± 2.94; p = 0.2). Improvement in BPR was only seen in non-obese patients (24.02 ± 20.07 vs 30.18 ± 21.93; p = 0.019). Improvement in functional variables occurred despite increase in BMI in non-obese (25.91 ± 2.85 vs 26.21 ± 2.96; p = 0.031), and there was no significant change in BMI in obese (35.30 ± 5.60 vs 34.93 ± 5.42; p > 0.05).

Conclusion

CR concurrently improves functional outcomes in both obese and non-obese patients despite no associated weight loss. The difference in BPR, however, is seen in only non-obese individuals. Future studies are needed to validate the role of weight-optimized CR protocols as a potential target for improving cardiac outcomes.

## Introduction

Cardiovascular disease (CVD) is a leading cause of morbidity and mortality and is responsible for approximately 20% of the worldwide disease burden [1]. Although the mortality from coronary heart disease has decreased over recent decades, nearly 470,000 recurrent myocardial infarctions are reported annually in the United States [2]. Cardiac rehabilitation (CR), a structured outpatient program, has become an essential component in the continuum of care post-hospital discharge to improve patient’s quality of life and social functioning and also as a means for secondary prevention of CVDs [3]. CR has been shown to decrease both CVD mortality and hospital readmissions by 25% and 18%, respectively [4]. Professional medical societal guidelines strongly recommend referral to CR after acute coronary syndrome, percutaneous coronary syndrome, coronary artery bypass surgery (CABG), stable angina, and peripheral arterial disease (class Ia recommendation) [5]. Despite the expansion of candidacy for CR to include a variety of cardiovascular conditions, it remains underutilized in the United States, with an estimated enrollment of only 10% to 20% of greater than two million eligible patients per year [6]. Although CR is a multidisciplinary intervention, weight loss programs have not been traditionally included in these secondary prevention programs [7]. As the prevalence of obesity has reached epidemic levels, with at least 40% of the patients with CVD having body mass index (BMI) ≥ 30 kg/m^2^, and also as obesity is a direct independent risk factor for CVD, it remains prudent to study the effects of CR in this patient population [8,9]. There are three phases of CR: initial phase, outpatient cardiac rehab, post-cardiac rehab. In this context, we studied the impact of CR on functional outcomes such as chronotropic competence (CC), heart rate (HR), and systolic blood pressure (SBP) in the obese patient population compared with the non-obese patient population.

## Materials and methods

Patient population

We performed a retrospective analysis of 178 patients undergoing CR at the West Virginia University Heart and Vascular Institute between July 2015 and September 2017. Patients had to complete at least phase I and 50% of phase II of CR and had at least three months of follow-up in the cardiology clinic following the last session of CR. Demographic and clinical variables were assessed, including age, sex, race, smoking, hypertension (HTN), hyperlipidemia (HLD), diabetes mellitus (DM), cerebrovascular accident (CVA), congestive heart failure (CHF), and ischemic heart disease. Patients were also assessed for use of medications including antiplatelet drugs, angiotensin-converting enzyme inhibitors (ACEIs), angiotensin II receptor blockers (ARBs), beta-blockers, statins, and other optimal medical therapy (OMT). OMT was defined as the use of aforementioned medications without any limitation. BMI-based screening was performed to classify the patients into non-obese (BMI < 30 kg/m^2^; n = 87) and obese (BMI ≥ 30 kg/m^2^; n = 91) (Table 1). Patients who had surgeries for CABG, valve disease, and left ventricular assist device, as well as non-surgical patients such as those with heart failure, post-percutaneous coronary intervention, and myocardial infarction were included.

**Table 1:** Demographics of patients undergoing cardiac rehabilitation BMI, body mass index; ACEI, angiotensin-converting enzyme inhibitor; ARB, angiotensin II receptor blocker; OMT, optimal medical therapy

	Non-obese (N = 87), BMI < 30 kg/m^2^	Obese (N = 91), BMI ≥ 30 kg/m^2^	p-Value
Age (years)	66.39 ± 11.27	64.37 ± 9.7	0.203
Gender (male), N (%)	62 (71.26 %)	64 (70.33 %)	0.891
Race (Caucasian), N (%)	83 (95.4 %)	88 (96.7 %)	0.655
Smoking (yes), N (%)	13 (14.94 %)	2 (2.2 %)	0.002
Hypertension (yes), N (%)	71 (81.61 %)	71 (78.02 %)	0.551
Hyperlipidemia (yes), N (%)	59 (67.82 %)	53 (58.24 %)	0.185
Diabetes mellitus (yes), N (%)	27 (31.03 %)	42 (46.15 %)	0.038
Cerebrovascular accident (yes), N (%)	8 (9.2 %)	7 (7.69 %)	0.718
Congestive heart failure (yes), N (%)	11 (12.64 %)	19 (20.88 %)	0.142
Ischemic (yes), N (%)	56 (74.67 %)	67 (81.71 %)	0.284
Antiplatelets (yes), N (%)	79 (90.8 %)	84 (92.31 %)	0.718
Beta-blockers (yes), N (%)	76 (87.36 %)	77 (84.62 %)	0.599
ACEIs/ARBs (yes), N (%)	45 (51.72 %)	61 (67.03 %)	0.037
Statins (yes), N (%)	66 (75.86 %)	77 (84.62 %)	0.141
OMT (yes), N (%)	29 (36.71 %)	51 (57.95 %)	0.006
BMI	25.91 ± 2.84	35.30 ± 5.60	< 0.001
Sessions attended (N)	27.00 ± 10.97	24.97 ± 11.59	0.231

Cardiac rehabilitation

Supervised exercise and numerous measurements and assessments were included in CR. In addition to restoring physical function, patients also received information and tools to change certain aspects of their lifestyle, such as smoking, nutrition, stress, and medications. Trained nurses and outpatient dieticians provided counseling for lifestyle and risk factor modification. Patients received vocational rehabilitation to return to work safely and promptly with severe obesity. The physical training program was performed under the supervision of a cardiologist and exercise physiologist as per the American Heart Association (AHA), American Association of Cardiovascular and Pulmonary Rehabilitation guidelines [10].

The exercise program included flexibility training by static stretching with an emphasis on the lower back and thigh, with intensity was to the point of mild discomfort, and aerobic exercise including walking, treadmill, and cycling, with intensity based on symptom-limited exercise test. Rate of perceived exertion (RPE) was 11-16, based on Borg 6-20 scale, with each session lasting for 15-60 minutes, three to five times per week. Strength training was achieved using dumbbells, free weights, and machine weights to the level of moderate fatigue, with an RPE of 11-13.

Symptom-limited exercise tests were performed while observing symptoms and monitoring HR, blood pressure (BP), RPE, electrocardiogram (ECG) progressing from continuous monitoring to intermittent as appropriate for the risk level of the patient.

We evaluated multiple functional variables known to impact cardiac mortality using a symptom-limited exercise stress test based on the standard or modified Bruce protocol. The following variables were assessed: cardiorespiratory fitness, predicted using a prediction equation (TM-VO2pred), METS, CC, and blood pressure response ([BPR] difference of SBP at baseline and peak exercise - delta SBP).

Assessment of these variables was done before and after the completion of CR, followed by subgroup analysis of change in BMI in non-obese (BMI < 30 kg/m^2^) and obese (BMI ≥ 30 kg/m^2^) patients. We also assessed whether functional outcomes were associated with weight loss. A pre- and post-CR BMI was calculated in obese and non-obese subgroups to look for statistical significance.

Statistical analysis

Normality assumption was tested, and paired tests for HR (CC), BPR (delta SBP), and METS were performed (Table 2). Wilcoxon signed-rank test was performed for pre- and post-means for each variable, with the paired test significance at p < 0.05.

**Table 2:** Pre and post cardiac rehabilitation functional outcomes ^£^pre vs post: p < 0.001; ^¥^pre vs post: p = 0.010; ^¢^pre vs post: p = 0.019; ^†^pre vs post: p = 0.031 BMI, body mass index; CC, chronotropic competence; BPR, blood pressure response; METS, metabolic equivalence

	Non-obese (N = 87), BMI < 30 kg/m^2^	Obese (N = 91), BMI ≥ 30 kg/m^2^	p-Value
Pre-CC	0.71 ± 0.11^£^	0.72 ± 0.10^¥^	0.867
Post-CC	0.76 ± 0.11^£^	0.75 ± 0.12^¥^	0.440
Pre-BPR	24.02 ± 20.07^¢^	27.18 ± 17.99	0.136
Post-BPR	30.18 ± 21.93^¢^	28.07 ± 20.12	0.527
Pre-METS	4.96 ± 1.98^£^	4.39 ± 1.81^£^	0.050
Post-METS	7.33 ± 2.94^£^	6.79 ± 3.34^£^	0.200
Pre-BMI	25.91 ± 2.85^†^	35.30 ± 5.60	<0.001
Post-BMI	26.21 ± 2.96^†^	34.93 ± 5.42	<0.001

## Results

Among 178 patients included in our study, 87 patients were non-obese and 91 patients were obese. Baseline demographic and clinical characteristics were well distributed for age, gender, race, HTN, HLD, DM, CVA, CHF, antiplatelet agents, ACEIs/ARBs, beta-blockers, and OMT (Table 1). More non-obese patients (n = 13; p = 0.002) continued to smoke at the time of completion. There was a higher prevalence of DM among obese compared with non-obese patients (n = 42; p = 0.038). Compared with non-obese patients, obese patients were more likely to use OMT (n = 51; p = 0.006) and ACEI/ARB (n = 61; p = 0.037). There was no significant difference between the number of sessions attended between the two groups (p = 0.23) (Table 1).

**Figure 1:**
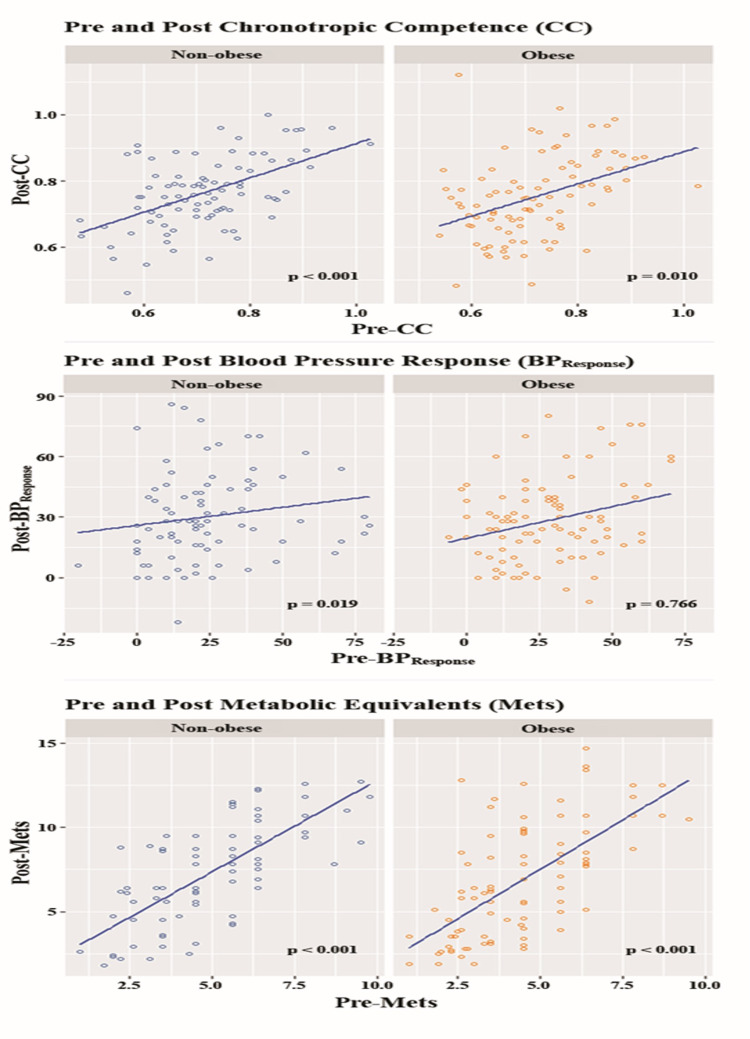
Scatter plot for functional outcomes in non-obese and obese patients undergoing cardiac rehabilitation.

The results showed improvement in cardiorespiratory fitness (non-obese: 4.96 ± 1.98 to 7.33 ± 2.94, p < 0.001; obese: 4.39 ± 1.81 to 6.79 ± 3.34, p < 0.001) and CC (non-obese: 0.71 ± 0.11 to 0.76 ± 0.11, p < 0.001; obese: 0.72 ± 0.10 to 0.75 ± 0.12, p = 0.010), whereas BPR only showed improvement in non-obese patients (24.02 ± 20.07 to 30.18 ± 21.93, p = 0.019) (Table 2). A scatter plot of the results of pre and post HR, BP, and METS response for each subgroup is presented in Figure 1. Weight loss was not a determinant for improved functional outcomes post-CR.

## Discussion

In this institutional study involving 178 patients who underwent CR post-hospital admission for various cardiovascular conditions, we found that cardiorespiratory fitness and CC improved regardless of the BMI. More importantly, there was no correlation between weight loss and improved functional outcomes.

CR is a multidisciplinary intervention that is aiming to optimize the physical, psychological, and social well-being of patients or even regression of underlying atherosclerotic processes to reduce cardiovascular morbidity and mortality [2]. It facilitates the ability of the patient to preserve or resume an active and functional lifestyle. CR has emerged as an effective avenue to deliver secondary cardiovascular prevention care [11]. As such, the AHA strongly recommends CR for all patients with CVDs. It is well known that functional outcomes such as CC, SBP response, and level of physical endurance impact cardiac morbidity and mortality in patients with heart disease [12-14]. Despite its beneficial effects, referral rates to CR post-cardiovascular procedures remain low in the United States. As such, professional medical societies proposed home-based CR as an alternative to increasing enrollment in CR [10].

Previous research demonstrated that CR improves exercise capacity and physical functioning in both home-based and center-based settings [10]. However, there are limited data to suggest whether such improvement in functional outcomes tends to occur irrespective of underlying BMI. Lim et al. demonstrated that enhancement in functional outcomes has occurred irrespective of BMI in the Korean patient population who suffered acute myocardial infraction and underwent CR [15]. As such, there are no contemporary studies in the American population. Obesity has become an epidemic in the United States, with an estimated 40% of persons aged 20 years having a BMI of 30 kg/m^2^ [8,9]. Severe obesity is an independent risk factor for CAD, HTN, heart failure, and arrhythmias, thereby significantly influencing CR outcomes [16-18].

We found that CR through its physical conditioning, strength training, and psychosocial risk factor modification tends to improve functional variables, which are directly related to cardiac outcomes irrespective of weight loss. There is an overall improvement in CC and cardiorespiratory fitness, whereas the SBP response continued to show a normal response of >20 mmHg with completion of CR. More than half of the patients who participated in our CR study were obese, with a mean BMI of 35.30 ± 5.60 kg/m^2^. This is similar to prior studies suggesting a higher prevalence of obese patients undergoing CR. This growing set of obese population behaved differently than non-obese patients only concerning SBP response. The improvement was observed despite <50% of patients being on OMT out of a cohort of patients where almost 70% of patients had ischemic heart disease. The fact that non-obese and obese patients behave differently in outcomes of BPR despite overall benefit in physical endurance and CC likely points toward different physiological subsets undergoing similar lifestyle risk factor modification. Whether this ultimately translates into a difference in morbidity and mortality outcomes in these patients is yet to be studied. Based on our study, weight loss is not the likely target for improving functional outcomes. In fact, weight gain and an increase in BMI are likely to occur as patients recover from underlying heart disease. Long-term follow-up of outcomes in CR patients based on BMI may unravel interesting results leading to changes in our approach toward the different protocols of CR being applied in these patients.

Study limitations

The limitation of our study is predominantly related to it being a retrospective, single-center observational study. In our study, the subgroup of obese patients showed a higher incidence of DM with more use of ACEIs/ARBs and OMT. The results were related to a short-term follow-up, with few patients dropping out before completion of phase II of CR. Whether outcomes continue to differ in long term with valid implications on cardiac morbidity and mortality need to be further studied.

## Conclusions

Retrospective analysis of functional outcomes including METS, HR, and BPR in those undergoing CR based on underlying BMI showed that CR improves functional outcomes in patients with heart disease in both obese and non-obese patients. This improvement tends to occur irrespective of weight loss and BMI. The difference in BP is seen only in non-obese patients. Further long-term studies on CR may help establish a weight-based protocol for improved cardiac functional outcomes.
